# Towards the elimination of malaria in South Africa: a review of surveillance data in Mutale Municipality, Limpopo Province, 2005 to 2010

**DOI:** 10.1186/1475-2875-12-7

**Published:** 2013-01-08

**Authors:** Ester Khosa, Lazarus R Kuonza, Phillip Kruger, Eric Maimela

**Affiliations:** 1 School of Health Systems and Public Health, Faculty of Health Sciences, University of Pretoria, Pretoria, South Africa; 2 South Africa Field Epidemiology and Laboratory Training Program, National Institute for Communicable Diseases of the National Health Laboratory Services, Private Bag X1, Sandringham, 2131, South Africa; 3 Department of Health, Limpopo Province, South Africa

**Keywords:** Malaria, Malaria incidence rate, Malaria case fatality rate, Indoor residual spraying coverage, Limpopo Province

## Abstract

**Background:**

South Africa has targeted to eliminate malaria by the year 2018. Constant monitoring of malaria morbidity and mortality trends in affected subpopulations is therefore crucial in guiding and refining control interventions. Mutale Municipality in Limpopo Province is one of the areas with the highest risk of malaria in the country. This paper describes trends in malaria incidence, case fatality and household indoor residual spraying (IRS) coverage in Mutale Municipality, during the period 2005 to 2010.

**Methods:**

A retrospective descriptive analysis was conducted on malaria data routinely collected through the Limpopo provincial malaria information system between July 2005 and June 2010. Five malaria seasons were defined. Annualized malaria incidence rates, case fatality rates (CFR) and IRS coverage rates were calculated.

**Results:**

Cumulatively, 4,663 malaria cases and 21 malaria deaths were reported in Mutale between July 2005 and June 2010. Investigation of likely origin of the malaria in 3,517 patients revealed that 6.6% were imported cases, mostly from neighbouring Zimbabwe (222/231). Malaria incidence rates fell from 13.6 cases per 1,000 person-years in the 2005–2006 season to 2.7 cases per 1,000 person-years in the 2009–2010 season. The mean malaria CFR was stable between 0.3 and 0.6% during the first four seasons, and increased sharply to 2.1% in the 2009–2010 season. The median age of the 21 malaria deaths was 34 years (range: 16 to 60 years). CFRs were 0% in children below 15 years and above 0.5% in patients more than 24 years old. Regular IRS achieved coverage above 80% in all five seasons.

**Conclusion:**

Malaria control interventions implemented in Mutale significantly reduced the incidence of malaria in the population. In order to accurately monitor progress towards the elimination goal, the malaria control programme should strengthen the reporting and capturing of the data in the provincial malaria information system; all patients diagnosed with malaria should be investigated to determine the likely source of the malaria, and malaria related deaths should be audited to improve case detection and management. Furthermore, the country should strengthen cross border malaria control collaborations in order to minimize malaria importation.

## Background

Malaria is a major public health concern in many parts of the world, especially in sub-Saharan Africa where about 81% of the global malaria cases and 90% of the deaths occur [1]. The disease is a leading cause of morbidity and mortality, mostly affecting young children (below five years of age) and pregnant women [1,2]. Recent reports demonstrate that malaria control can be achieved in several countries by scaling up existing proven interventions possibly towards elimination or complete interruption of remaining residual foci at a local level [3-6]. Pivotal to achievement of these objectives are effective malaria surveillance systems providing information on the number and distribution of malaria cases and deaths for targeted and intensified control efforts [4,6].

South Africa is a low malaria transmission setting characterized by lower incidence of confirmed cases. The country has a well-established malaria control programme, and has implemented malaria control interventions since the 1930s [7]. Malaria transmission in the country has decreased over the years, and is now limited to the low-lying, north-eastern parts of Limpopo, Mpumalanga and KwaZulu-Natal Provinces, along the borders with Zimbabwe and Mozambique. In these areas malaria transmission is mostly seasonal, unstable and epidemic prone [8].

Over the past decade, Limpopo Province has reported the largest proportion of the malaria cases diagnosed in South Africa each year [9]. The majority of the cases have been diagnosed in Mutale Local Municipality, which is located at the north-eastern border of Vhembe District, along the border with Zimbabwe and Mozambique [8,9].

South Africa has set a target to eliminate local mosquito-borne transmission of malaria by the year 2018. Key milestones within the malaria elimination continuum include four phases (1) control to less than 5 positive cases per 1000 persons presenting with fever to transition to (2) pre-elimination, to less than one case per 1000 persons at risk per year to transition to (3) elimination and (4) prevention of re-introduction of malaria, both which aims at zero cases per 1000 persons at risk per year [4,10]. Accordingly, as the country moves forward with the malaria elimination efforts, it is crucial to monitor trends in morbidity and mortality due to malaria in the affected populations, in order to guide and refine control interventions. The current paper describes trends in malaria incidence, case fatality and household indoor residual spraying (IRS) coverage in Mutale Local Municipality, using historical data routinely collected by the Department of Health in Limpopo Province between July 2005 and June 2010.

## Methods

### Study setting and design

**Figure 1:**
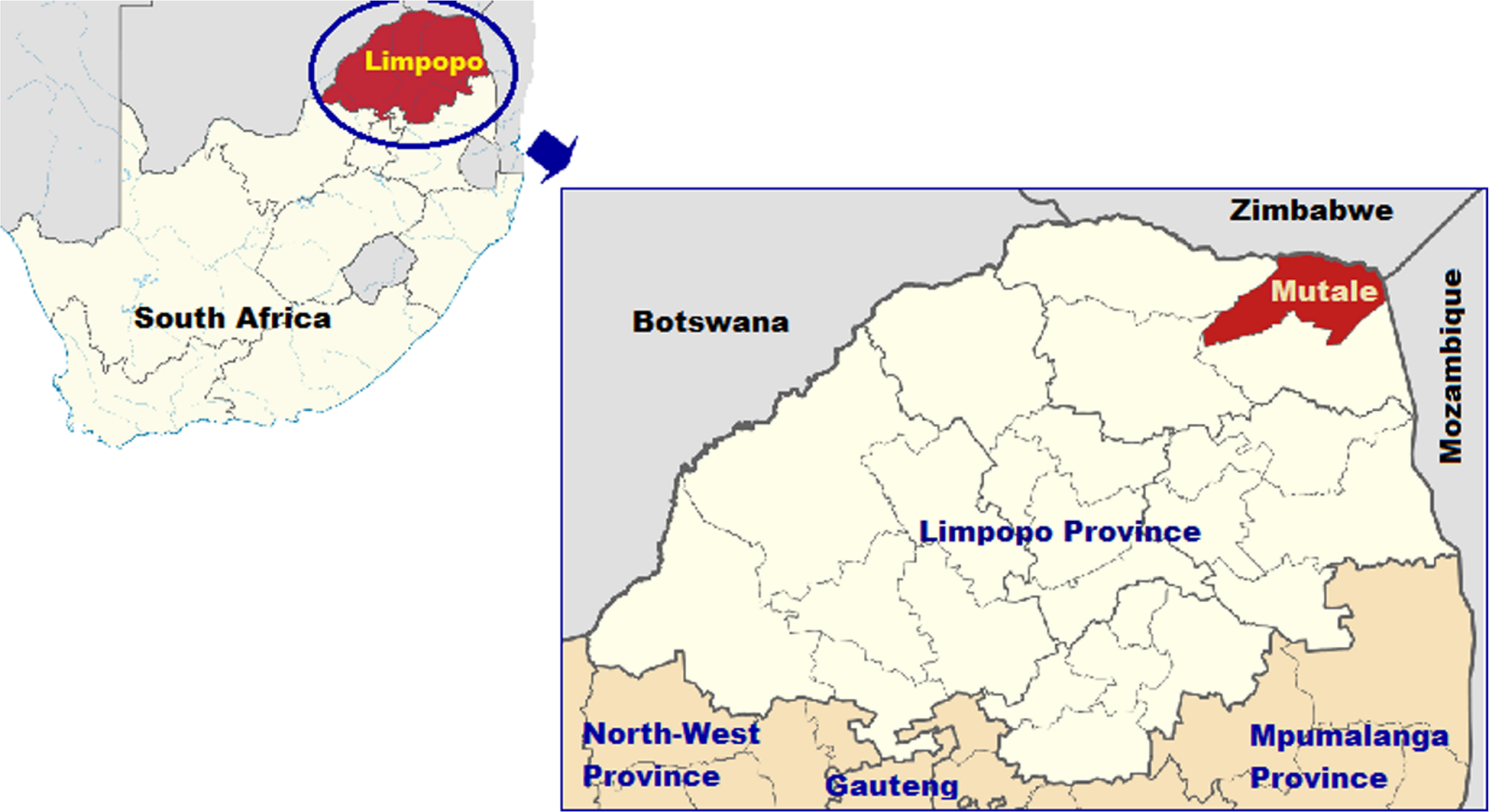
**Map showing the geographical location of Limpopo Province in South Africa and Mutale Local Municipality in Limpopo Province.** The top insert shows the location of Limpopo Province in South Africa (in blue circle). The bigger map shows the location of Mutale Municipality in Limpopo Province, in relation to neighbouring countries of Zimbabwe and Mozambique. The map was adapted from the Wikipedia website [http://en.wikipedia.org/wiki/File:Map_of_Limpopo_with_Mutale_highlighted_(2011).svg] as an open source document published under the GNU Free Documentation License and the copyright holder grants permission to copy, distribute and/or modify the document.

Mutale is one of the four municipalities in Vhembe District of Limpopo Province. Situated in the far north-eastern corner of South Africa, bordering with Zimbabwe and Mozambique, Mutale is mostly rural and has an estimated population of just over 108,000 inhabitants (2011 estimates from the District Health Information System). Over the past decade the municipality has constantly reported the highest malaria incidences in Limpopo Province, with transmission occurring mostly during the rainy season, between December and May [11]. Figure 1 shows the geographical location of Mutale Municipality in Limpopo Province and in South Africa.

A retrospective descriptive analysis was conducted on historical malaria data routinely collected through the Limpopo provincial malaria information system. The data included malaria case patients and malaria deaths reported from health facilities in Mutale between July 2005 and June 2010, and malaria household IRS data captured by the provincial malaria control programme during the same period.

### The malaria information system in Limpopo province

Malaria is a notifiable disease in South Africa in terms of the National Health Act [12]. When a patient presents to a health facility with signs and symptoms of malaria, the attending health worker collects a blood specimen for parasitological confirmation (using blood smear microscopy or rapid diagnostic testing). When the patient tests positive for malaria the health worker completes a malaria case notification form and reports the patient to the provincial malaria control programme. The case report form collects patient demographic information, clinical information, pregnancy status (for female patients), travel history and possible place of infection.

Malaria case investigators from the malaria control programme visit each health facility on a weekly basis to verify the malaria case reports. The case investigator then visits the household of each reported malaria case to conduct a full epidemiological investigation and contact tracing. All case-patients who test positive for malaria during active contact tracing are also reported through the malaria information system. The malaria case report forms are then sent to the provincial malaria control programme (situated in Tzaneen), where the reports from the whole province are captured electronically into a Microsoft Access® database. Private health facilities are also required to report all malaria-positive case-patients diagnosed and treated in the respective facilities.

### Malaria vector control in Limpopo province using indoor residual spraying

Indoor residual spraying (IRS) is the primary vector control intervention used to reduce malaria transmission in Limpopo Province. IRS involves the application of long-acting chemical insecticides onto the inside walls and roofs of dwellings of people living in areas where there is a high risk of malaria transmission, in order to kill the adult vector mosquitoes that transmit the malaria parasites. In Limpopo Province IRS is conducted annually, in all the low-altitude, high malaria transmission areas within the province (including Mutale). Each spraying season is scheduled to begin in August and end in March the following year, in line with the malaria transmission season in the province. The malaria control programme uses two chemical insecticides for malaria vector control, dichloro-diphenyl-trichloroethane (commonly known as DDT) and a synthetic pyrethroid approved by the WHO for malaria vector control. DDT is used to spray walls made from porous materials such as mud or wood, and pyrethroids are used to spray plastered or painted walls [13,14]. When conducting IRS, the spraying teams record the number of household structures sprayed (and those visited but not sprayed) as well as the amount (in kilograms) and type of insecticide used on a daily basis [14,15]. This information is sent to the provincial malaria control programme where it is captured electronically into a Microsoft Access® database.

### Data analysis

Data were extracted from the provincial Microsoft Access® database onto a Microsoft Excel® spreadsheet for cleaning and analysis. The data was later imported into the EpiInfo statistical software (version 3.5.1, Centers for Disease Control and Prevention, Atlanta, USA) for simple descriptive analysis. Since malaria transmission in Mutale is seasonal, the data were analysed and presented using the traditional malaria seasons in Limpopo Province. For the purposes of this study, a malaria season was defined as the period from 1 July to 30 June the following year.

In calculating the malaria incidence (per 1,000 population at risk per year), the number of reported malaria cases for each season was divided by the corresponding average mid-season population (calculated as a mean of the mid-year population estimates of the current year and the following year). The mid-year population estimates were obtained from the District Health Information System (DHIS). Malaria cases that were reported as imported malaria and those that were untraceable were excluded from the incidence calculations (these were assumed to have been infected outside the municipality). Malaria case fatality rate (CFR) was defined as the number of malaria deaths reported in a malaria season divided by the total number of malaria cases that were reported during that season. The CFRs were expressed as percentages (ie., number of deaths per 100 malaria cases). In calculating IRS coverage, the total number of household structures sprayed during a malaria season was divided by the total number of sprayable structures that had been targeted during that season, expressed as a percentage. Graphs and tables were created to illustrate the trends in malaria incidence, CFRs and IRS coverage during the five-year period.

### Ethical considerations

Ethical clearance was provided by the Faculty of Health Sciences Research Ethics Committee of the University of Pretoria (protocol number: S108/2011). All data analyzed in the study were extracted from the Provincial malaria information system, no further information was sought from the patients.

## Results

In total, 4,663 malaria cases were diagnosed in Mutale between July 2005 and June 2010. Out of these, 3,181 (68.2%) were captured as locally transmitted malaria, 231 (5%) were imported malaria, 105 (2.3%) untraceable, and the rest, 1,146 (24.6%), had no classification on the source of the malaria.

Table 1 shows the distribution of the diagnosed malaria cases and their origin by malaria season. Malaria cases in Mutale peaked during the 2007–2008 malaria season, before decreasing gradually in the subsequent two seasons. Of the 231 cases recorded as imported malaria, the majority (96.1%) were imported from Zimbabwe, and the rest were imported from Mozambique (seven cases), Malawi (one case) and Zambia (one case).

**Table 1:** Distribution of diagnosed malaria cases and their origin by malaria season, Mutale Municipality, Limpopo province, July 2005 to June 2010

**Malaria season**	**Origin of malaria**				**Total**
	**Local**	**Imported**	**Untraceable**^**§**^	**Not documented**^**α**^	
2005-2006	947	110	57	213	1327
2006-2007	595	44	15	61	715
2007-2008	1216	43	9	258	1526
2008-2009	208	23	10	590	831
2009-2010	215	11	14	24	264
Total	3181	231	105	1146	4663

^§^Untraceable cases are those that were investigated/followed-up by the malaria case investigators but the patients could not be located.

^α^For undocumented cases, there was no information recorded to indicate whether the patient was investigated/followed-up or not.

**Figure 2:**
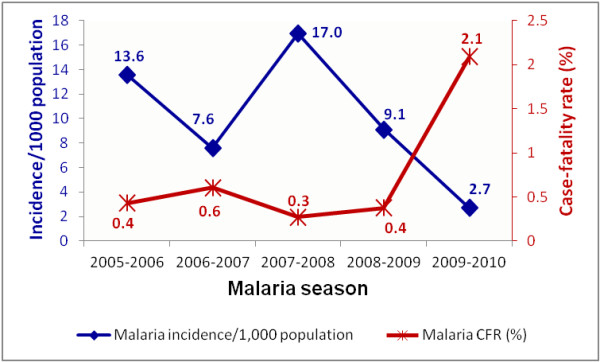
**Distribution of malaria incidence rates and case fatality rates per malaria season, Mutale Municipality, Limpopo Province, July 2005 to June 2010.** The blue line graph shows the incidence of malaria per 1,000 persons at risk each malaria season. The red line graph is showing the malaria case fatality rate (percentage) for each malaria season. Incidence calculations excluded imported and untraceable malaria cases (these were assumed to have been infected out of Mutale).

Figure 2 shows the distribution of malaria incidence rates and case fatality rates in Mutale Municipality per malaria season. Apart from the 2007–2008 malaria season, the annual malaria incidence rates in Mutale decrease from 13.6 cases per 1,000 population in the 2005–2006 season to 2.7 cases per 1,000 population in the 2009–2010 season. During the 2007–2008 malaria season the malaria incidence rate surged to 17.0 cases/1,000 population at risk. Twenty-one malaria deaths were recorded in Mutale during the period, indicating a mean case fatality rate of 0.5% per malaria season. The malaria case fatality rates were fairly stable (between 0.3% and 0.6%) during the first four seasons, and increased sharply to 2.1% during the 2009–2010 season.

Fifty-seven malaria cases had no record of age. The median age of the case-patients was 23 years (IQR = 13 to 36 years, ranging from less than a year to 99 years). The median age of the 21 malaria deaths was 34 years (IQR = 32 to 51 years, range 16 to 60 years). Table 2 shows the distribution of the mean malaria incidence rates and case fatality rates in Mutale for the five malaria seasons by age categories. The mean malaria incidence rates were highest among people aged between 25 and 44 years. Throughout the five malaria seasons Mutale municipality did not report any malaria deaths in children below 15 years of age. The malaria case fatality rates were above 0.5% in people above 25 years of age.

**Table 2:** Malaria incidence rates and case fatality rates by age category, Mutale Municipality, July 2005 to June 2010

**Age group category**	**Number of cases**^**§**^	**Number of deaths**	**Incidence rate/1,000 population per year**^**§**^		**Case fatality rate/100 malaria cases**^**§**^	
			**Rate**	(95% CI)^*^	**Rate**	(95% CI)*
0-4	336	0	5.82	*(5.22-6.47)*	0	*-*
5-14	940	0	7.74	*(7.26-8.24)*	0	*-*
15-24	1072	1	10.95	*(10.31-11.62)*	0.08	*(0.004-0.41)*
25-34	796	10	13.34	*(12.46-14.30)*	1.11	*(0.57-1.94)*
35-44	470	3	13.46	*(12.29-14.70)*	0.60	*(0.15-1.60)*
> 44	656	7	10.50	*(9.73-11.32)*	1.03	*(0.32-1.43)*
Total	4270	21	9.84	*(9.55-10.14)*	0.50	*(0.29-0.68)*

^§^ Number of cases, incidence rates and case-fatality rates exclude imported and untraceable malaria cases and another 57 cases that had no record of age. * CI = Confidence Interval.

**Figure 3:**
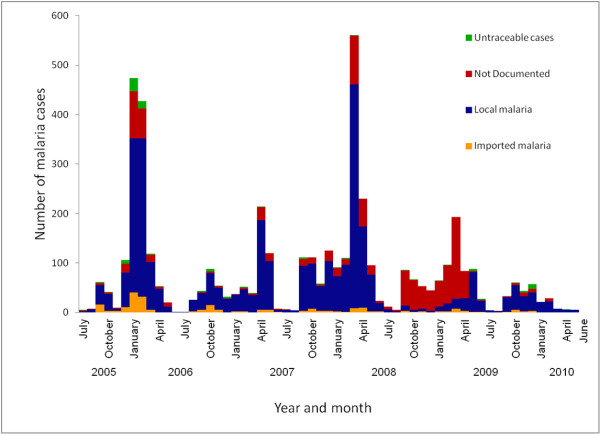
**Number of malaria cases by month and year of diagnosis, Mutale Municipality, Limpopo Province, July 2005 to June 2010.** Untraceable cases are those that were investigated/followed up by the malaria case investigators but the patients could not be located. For undocumented cases there was no information recorded to indicate whether the patient was investigated/followed up or not.

Figure 3 shows the seasonal variation in the transmission of malaria in Mutale during the five-year study period. Malaria transmission occurred mostly from September to May, and the cases became very low during the winter months of June and July. The peak incidences decreased gradually during the first two seasons (2005 to 2007), rose sharply during March 2008 and decreased in the last two seasons (2008 to 2010). The number of imported malaria cases also decreased steadily from 110 cases during the 2005–2006 season, to 11 cases during the 2009–2010 season. Of the 831 malaria cases reported during the 2008–2009 season, 590 (71%) had no documentation of the source of the malaria.

Table 3 shows the total number of household structures sprayed during each IRS season and the IRS coverage for each season. The malaria control programme achieved IRS coverage rates above 90% in four of the five seasons. During the 2006–2007 malaria season the IRS coverage rate was 79%, which was below the recommended minimum target of 85%, while during the 2007–2008 malaria season the IRS coverage was above 100%.

**Table 3:** Number of household structures sprayed and the IRS coverage per spraying season, Mutale Municipality, July 2005 to June 2010

**Malaria season**	**Household structures targeted for IRS**	**Achieved IRS coverage**	
		**n**	**%**
2005-2006	56 340	55 448	98.4
2006-2007	60 213	47 806	79.4
2007-2008	60 989	63 291	104.3
2008-2009	63 580	58 936	92.7
2009-2010	65 490	63 020	96.2
Total	306 612	308501	94.2

*IRS = Indoor residual spraying.

## Discussion

The findings demonstrated an overall decreasing trend in malaria morbidity in Mutale during most seasons of the study period, most remarkebly after a peak during the 2007/08 season. The decreasing trend is in line with patterns that have been reported elsewhere in Limpopo Province and in other malarious provinces in South Africa [9,16]. The decrease has been attributed to the synergistic effect of the scaling up of IRS in the country and the introduction of artemisinin-based combination therapy (ACT) for the treatment of uncomplicated malaria in 2004 [17]. Implementation of widespread use of ACT in the treatment of malaria has been shown to directly decrease malaria transmission, in addition to improving the malaria cure rates [18-20].

Furthermore, the implementation of the tri-national Lubombo Malaria Protocol as part of the Lubombo Spatial Development Initiative (LSDI) aimed at enhancing economic development in the Lubombo region (a mountainous region shared by South Africa, Swaziland and Mozambique) probably contributed to the decline of malaria in Mutale [21,22]. Although Mutale does not directly fall within the Lubombo region, the municipality significantly benefited from the reduction of malaria in neighbouring countries such as Mozambique as a result of the LSDI malaria control efforts. Findings from this study seem to support this view since only a small proportion of the imported malaria cases were from Mozambique despite Mutale being situated along her border, and the majority of the imported cases were from Zimbabwe which was not part of the LSDI. The LSDI initiative has also been complimented for reducing the burden of malaria in areas neighbouring the Lubombo region in southern Mozambique and in Swaziland [21].

Interestingly Mutale municipality experienced a surge in the incidence of malaria during the 2007–2008 season. Despite the high IRS coverage achieved during this season (exceeding 100%), Limpopo Province recorded an outbreak of malaria from March through April 2008. The outbreak was related to excessive rainfall recorded in the province from January to March 2008. Excessive rainfall has been shown to increase malaria transmission in various ways. Firstly, the increased rainfall provides more breeding pools for mosquitoes leading to an increased population of the malaria vector (*Anopheles arabiensis*) [23,24]. Secondly, the high temperatures and humidity often associated with high rainfall, compel people to spend the nights in cooler open areas outside the houses (or with open windows) thus further exposing them to the malaria vectors. Thirdly, prolonged heavy rainfall interrupts the IRS programme by disrupting the movement of the teams that will be conducting IRS, as some communities become inaccessible due to bad roads and possibly flooding. Consequently, the reported high IRS coverage (of 104%) possibly resulted from mop-up spraying of households in response to the outbreak, which normally includes re-spraying of some of the previously sprayed households.

One of the targets of the malaria control programme in South Africa is to maintain malaria case fatality rates (CFR) below 0.5% [10]. The present study revealed that malaria case fatality rates in Mutale were high in the older population (above 25 years of age). In a study conducted in Limpopo Province in 2008, Gerritsen and colleagues reported higher malaria CFRs in the older population, and they attributed this to the possibility of more severe malaria in the elderly and also the possibly of poor health-seeking behaviour in this age group [16]. However the study findings are in contrast to figures reported in most developing countries with high malaria endemicity, where the majority of malaria deaths occur in children below the age of five years [1]. According to statistics released by the Global Malaria Action Plan in 2009, at least 85% of malaria deaths that occurred globally in 2008 were in children below the age of five years [1].

Mutale maintained fairly high IRS coverage throughout the study period. However further enquiry on how the province derives IRS targets revealed that the actual population of household structures in Mutale municipality is not documented. When the malaria control programme is planning the IRS season the programme managers estimate the targeted structures based on the number of household structures that were sprayed in the previous season. This method of estimating the number of structures targeted for spraying does not take into account the actual population at risk of malaria. IRS for malaria control is a method for community protection, and to be effective, implementation has to be targeted carefully, treating only where and when necessary [14,17]. According to WHO guidelines [25], planning for an IRS season should include accurate estimation of the total population and determination of the total number of rooms that are used for sleeping or relaxing at night in the target area. This enables the programme managers to calculate the proportion of the population and the proportion of rooms they managed to protect through the IRS efforts at the community level, which are very useful process indicators for monitoring the performance of the IRS programme. The malaria control programme should work closely with the local community leaders in order to obtain accurate figures for the population figures at community level.

The decreasing malaria incidence in Mutale suggests that the malaria control efforts implemented during the period under study have been fairly effective. If the declining trend is sustained, it is feasible that Limpopo Province will soon be entering the pre-elimination phase of malaria. When a country/province/district is transitioning into the so-called “pre-elimination” phase of malaria, the WHO recommends a re-orientation of the malaria programme, which should be characterized by the adoption of more rigorous surveillance strategies in order to accurately measure and document the malaria incidence rates [4,6]. Accordingly, the malaria control programme in Limpopo should strengthen the surveillance, reporting and the capturing of data in the provincial malaria information system. This is especially important for areas like Mutale Municipality where there is high potential for imported malaria through the porous borders with Zimbabwe and Mozambique, countries that remain highly malaria endemic. The surveillance strategy should therefore ensure that all diagnosed malaria cases are investigated to determine the source of the malaria, in order to accurately monitor local transmission and document transmission due to importation. The malaria control programme should also ensure regular update of the denominator data required to compute rates of malaria morbidity and mortality as well as other programmatic indicators.

Several limitations should be taken into account when interpreting the findings from this study. Firstly, 24% of the malaria cases had no documentation of the location where malaria was presumably contracted. Some of these could have been people who came for treatment from outside Mutale, and thus could have resulted in an overestimation of the true incidence of malaria in Mutale. Secondly, though the population of Mutale is administratively divided into villages, village-specific population figures could not be obtained, and thus the study could not calculate the malaria incidences at the sub-municipality level to determine the problematic areas in terms of the high risk of malaria transmission. Similarly the number of household structures per village could not be obtained; therefore the study could not determine the IRS coverage down to the village level. The findings would have been more useful if the analysis had managed to map the malaria incidence rates and IRS coverage in Mutale at sub-municipality level. Thirdly, the data used in this study is routinely collected through the provincial malaria surveillance system, which relies mostly on passive reporting. It is therefore possible that some malaria cases (or deaths) could have been missed by the system (because of non-reporting or misdiagnoses), thereby underestimating the true incidence of malaria (or the malaria CFRs). However there were no changes in the reporting requirements for malaria during the period under study, it is therefore unlikely that any cases missed by the system could have affected the observed trends.

## Conclusion

Malaria incidence has decreased significantly in Mutale municipality, probably due to interventions that have been implemented in the area during the period of study. As Limpopo Province moves forward with the malaria elimination efforts, it is important for the malaria control programme to further tighten the malaria control interventions in order to effectively interrupt local malaria transmission. The malaria control programme in Limpopo should strengthen the surveillance, reporting and the capturing of data in the provincial malaria information system. All patients diagnosed with malaria should be thoroughly investigated to determine the likely source of the malaria, (ie., whether locally transmitted or imported). Furthermore, in order for the malaria elimination to be sustained South Africa should establish or strengthen cross border malaria control collaborations beyond Mozambique and Swaziland, in order to minimize malaria importation. This can be achieved through creation of malaria buffer zones in Southern Africa (and later pushing them beyond the sub-region), and developing an operational malaria early warning systems for early detection and prevention of localized epidemics.

## Abbreviations

ACT: Artemisinin-based combination therapy; CFR: Case fatality rate; DDT: Dichloro-diphenyl-trichloroethane; DHIS: District Health Information System; IQR: Inter-quartile range; IRS: Indoor residual spraying; LSDI: Lubombo Spatial Development Initiative; SAFELTP: South African Field Epidemiology and Laboratory Training Programme; WHO: World Health Organization.

## Competing interests

The authors declare that they have no competing interests.

## Authors’ contributions

EK contributed to the conception, design, analysis, interpretation of data, writing the manuscript and overall co-ordination of the study. LK contributed to the conception, design, analysis and interpretation of data, writing the manuscript and overall technical supervision of the project. PK contributed to the conception, data interpretation and revision of the manuscript. EM contributed to the conception of the study, data interpretation and the revision of the manuscript. All authors have read and approved this manuscript.
